# Rare Modifier Variants Alter the Severity of Cardiovascular Disease in Pseudoxanthoma Elasticum: Identification of Novel Candidate Modifier Genes and Disease Pathways Through Mixture of Effects Analysis

**DOI:** 10.3389/fcell.2021.612581

**Published:** 2021-06-08

**Authors:** Eva Y. G. De Vilder, Ludovic Martin, Georges Lefthériotis, Paul Coucke, Filip Van Nieuwerburgh, Olivier M. Vanakker

**Affiliations:** ^1^Center for Medical Genetics, Ghent University Hospital, Ghent, Belgium; ^2^The Research Foundation – Flanders, Ghent, Belgium; ^3^Department of Ophthalmology, Ghent University Hospital, Ghent, Belgium; ^4^Department of Dermatology, Angers University Hospital, Angers, France; ^5^Department of Vascular Physiology and Sports Medicine, Angers University, Angers, France; ^6^Department of Biomolecular Medicine, Ghent University, Ghent, Belgium; ^7^Department of Pharmaceutics, Laboratory of Pharmaceutical Biotechnology, Ghent University, Ghent, Belgium

**Keywords:** pseudoxanthoma elasticum, ABCC6, candidate modifier gene, cardiovascular disease, mixture of effects analysis, C-alpha test, SKAT-O

## Abstract

**Introduction:** Pseudoxanthoma elasticum (PXE), an ectopic mineralization disorder caused by pathogenic *ABCC6* variants, is characterized by skin, ocular and cardiovascular (CV) symptoms. Due to striking phenotypic variability without genotype-phenotype correlations, modifier genes are thought to play a role in disease variability. In this study, we evaluated the collective modifying effect of rare variants on the cardiovascular phenotype of PXE.

**Materials and Methods:** Mixed effects of rare variants were assessed by Whole Exome Sequencing in 11 PXE patients with an extreme CV phenotype (mild/severe). Statistical analysis (SKAT-O and C-alpha testing) was performed to identify new modifier genes for the CV PXE phenotype and enrichment analysis for genes significantly associated with the severe cohort was used to evaluate pathway and gene ontology features.

**Results** Respectively 16 (SKAT-O) and 74 (C-alpha) genes were significantly associated to the severe cohort. Top significant genes could be stratified in 3 groups–calcium homeostasis, association with vascular disease and induction of apoptosis. Comparative analysis of both analyses led to prioritization of four genes (*NLRP1*, *SELE*, *TRPV1*, and *CSF1R*), all signaling through IL-1B.

**Conclusion** This study explored for the first time the cumulative effect of rare variants on the severity of cardiovascular disease in PXE, leading to a panel of novel candidate modifier genes and disease pathways. Though further validation is essential, this panel may aid in risk stratification and genetic counseling of PXE patients and will help to gain new insights in the PXE pathophysiology.

## Introduction

Pseudoxanthoma elasticum (PXE, OMIM#264800) is an autosomal recessive ectopic mineralization disorder, characterized by elastic fiber mineralization in the mid-dermis of the skin, the elastic media of blood vessels and the Bruch’s membrane in the eye. The main vascular feature of the disease is medial calcification, leading to peripheral artery disease and narrowing of arteries of mainly the lower limbs for which surgical intervention may be necessary in advanced stages (Leftheriotis et al., 2013).

PXE is caused by bi-allelic pathogenic variants in the *ABCC6* gene (ATP binding cassette, subfamily C, member 6, OMIM^∗^603234), encoding an ATP-dependent transmembrane transporter. To date, over 300 different potentially pathogenic variants have been reported (Vanakker et al., 2008; Finger et al., 2009). Pathogenic variants in the *ENPP1* gene (ectonucleotide pyrophosphatase/phosphodiesterase 1; OMIM^∗^173335), the gene associated with Generalized Arterial Calcification of Infancy (GACI; OMIM#208000), were also found to cause PXE, although less frequently (Nitschke et al., 2012).

The pathophysiology of PXE is incompletely understood, but holds an important role for the calcification inhibitor inorganic pyrophosphate (PPi). The production of PPi relies on hydrolysis of ATP by the ENPP1 enzyme, after is has been transported from the liver to the vasculature. PPi serum levels are considerably decreased in PXE patients and ABCC6 deficiency has been demonstrated to hamper cellular ATP release, hence hindering PPi production (Jansen et al., 2014). Nonetheless, it is known that a lack of PPi is not the only disease mechanism in PXE. Also mitochondrial dysfunction and oxidative stress, apoptosis, inflammation and dysfunctional inhibitors of calcification, such as matrix gla protein (MGP) and fetuin-A have been described to contribute to the elastic fiber calcification in PXE patients, cell- and animal models (Uitto et al., 2010; Hosen et al., 2014; Brampton et al., 2021).

Striking for PXE is an extensive inter- and intrafamilial clinical variability, with no strong underlying genotype-phenotype correlations. Indeed, patients with the same pathogenic *ABCC6* variants may develop a different phenotype with respect to the type and severity of the symptoms. Moreover, identical pathogenic variants in *ABCC6* or *ENPP1* were shown to cause either PXE or GACI in different patients (Nitschke et al., 2012). All these findings suggest that other factors must contribute to the clinical variability in PXE besides the causal genes, e.g., environmental factors such as life-style, epigenetic modifications and modifier genes (Stitziel et al., 2011; Kwon et al., 2020). Different approaches could be followed for genetic modifier discovery. One approach is the single-marker test, whereby individual variants within a gene are tested for an association with the disease outcome, with standard univariate statistical tests, controlled by a multiple-comparison correction. Unfortunately, for this approach large groups are needed to obtain statistically valid results, especially when using whole exome sequencing (Zarbock et al., 2009). In PXE, single-marker tests were used to identify single nucleotide polymorphisms (SNPs) in the *VEGFA* gene as modifiers of the severity of the ocular phenotype, using a candidate gene approach (Boraldi et al., 2014; De Vilder et al., 2020). Further, the allelic variant epsilon2/epsilon3 of the *APOE* gene was suggested as a modifying factor for the cardiovascular PXE phenotype with a seemingly protective effect in older age (Clarke et al., 2013).

Contrary to modifiers with a high penetrance, it is assumed that multiple rare variants of low penetrance either within or across genes collectively may influence complex human traits, with a combined protective, deleterious or null effect. While the effects of each individual rare variant can be modest, collectively they can sufficiently increase or decrease the disease risk or severity (Konigorski et al., 2017). To identify new modifier genes, a multiple-marker test can be used, which tests multiple variants simultaneously with the use of multivariate methods (Lee et al., 2012). Examples of such tests are the classical burden tests, which have an optimal result when the direction of effect is the same for all assessed variants (Lee et al., 2012). However, this is not always the case, therefore testing for the presence of a mixture of effects of multiple rare variants on a trait may prove beneficial, for which the C-alpha and the (optimized) sequence Kernel Association (SKAT-O) test can be used (Emond et al., 2012; Konigorski et al., 2017).

Here, we implemented extreme phenotype sampling, in which individuals who are at both ends of the phenotypic distribution are analyzed. The rationale for this approach is that it can be assumed that the frequency of alleles that contribute to the trait are enriched in patients with an extreme phenotype, hereby improving the power of the analysis. Especially in the setting of rare diseases, where limited sample sizes are available, such an approach helps to obtain valid results (Lanktree et al., 2010; Emond et al., 2015). For example, in cystic fibrosis (CF; OMIM#219700), this approach was used to successfully identify several disease modifiers (Emond et al., 2015).

In this study, we evaluated the collective modifying effect of rare variants on the cardiovascular phenotype of PXE. For this, we performed whole exome sequencing in 11 PXE patients with an extremely mild or severe cardiovascular phenotype, based on clinical severity and Agatston score of the lower limbs. Subsequently, we performed SKAT-O and C-alpha tests to help identify modifiers of the cardiovascular disease severity, leading to a panel of novel candidate modifier genes and disease pathways.

## Materials and Methods

### Patient Characteristics

All patients were diagnosed with PXE based on a combination of skin pathology (elastic fiber mineralization and fragmentation in the mid-dermis of lesional skin, using Verhoef-van Giesson and Von Kossa staining), and typical (sub)retinal abnormalities on fundoscopy. Further, a molecular analysis of the *ABCC6* gene was performed, while variants in the *ENPP1* and *GGCX* gene were excluded (Hosen et al., 2015). Dichotomization of the patients into a severely vs. mildly affected group was based on calcium load of the vasculature of the lower limbs using the Agatston score following whole body CT scan: an Agatston score of > 1,000 was considered severe and < 200 was considered mild. Informed consent was obtained from all patients. Ethics Committee approval was obtained. Patient records were consulted only to obtain relevant patient data. The described research adhered to the tenets of the Declaration of Helsinki.

### Whole Exome Sequencing

Exome capture was performed according to the manufacturer’s protocol using the SeqCap EZ Human Exome Library v3.0 kit (Roche/Nimblegen, Madison, WI). After ligation of the barcoded Illumina (Eindhoven, The Netherlands) adapters, samples were pooled per 2 before capturing. Consequently, 6 samples were sequenced per Illumina HiSeq 2500 flow cell lane. On average 50E^6^ 2 × 100 bp paired end reads were sequenced per sample. On average, the mean coverage on the targeted exome bases was 37x across samples.

### Variant Calling

The Illumina sequencing reads were mapped against the human reference genome (hs37d5) using bwa (alignment via Burrows-Wheeler transformation) version 0.7.12-r1039 (Li and Durbin, 2009). Variants were called using GATK version 3.3-0-g37228af following the “best practice guidelines” from GATK (DePristo et al., 2011). Basically, this entails consecutive use of HaplotypeCaller, GenotypeGVCFs, V ariantRecalibrator and ApplyRecalibration. Variants were also called using freebayes v0.9.20-16-g3e35e72 after pre- processing the reads using Picard tools version 1.119^1^ for sorting and marking of duplicate reads and GATK version 3.3-0-g37228af (RealignerTargetCreator, IndelRealigner and BaseRecalibrator). The variants called by GATK and freebayes were merged using GATK CombineVariants. The merged results completely confirmed the SNP and small indel results previously reported by Hosen et al. (2015). The merged variant files were used to discover modifier genes in the “mild” and “severe” patient group. Larger indels were called separately using FishingCNV (Shi and Majewski, 2013) and ExomeDepth (Plagnol et al., 2012), confirming the exon 23–24 deletion in patient P2 previously reported by Hosen et al. (2015).

### Annotation Based Filtering

wANNOVAR (Chang and Wang, 2012) was used to annotate functional consequences of genetic variants. The tool was also used to filter the variants based on the “rare recessive disease model” option provided by wANNOVAR. This option filters variants that do not meet the requirement that more than two deleterious alleles (compound heterozygous or homozygous or hemizygous in chrX in males) need to be found in the same gene. wANNOVAR identifies these possible deleterious variants by selecting splicing and exonic variants that change protein coding, by removing variants observed in 1,000 Genomes Project, NHLBI- ESP 5400 exomes and dbSNP with a high minor allele frequency (≥ 5%), and by assessing the deleterious effect of the amino acid change using different methods such as SIFT (Ng and Henikoff, 2003) and Polyphen2 (Adzhubei et al., 2010).

### Statistics

SKAT-O statistics were calculated using the SNP-Set (Sequence) Kernel Association Test, originally developed by Wu et al. (2011). The test is implemented and maintained in an R package “SKAT” by Seunggeun Lee. This R package is in turn integrated in the Variant Association Tools (VAT) package. The method aggregates individual SNP score statistics in a SNP set and efficiently computes SNP-set level *p*-values. The SKAT-O statistics were calculated using all variants with types: “non-synonymous,” “splicing,” “stoploss,” “stopgain” or “ncRNA” (using VAT). Only genes with *p* < 0.005 are reported.

C-alpha statistics were calculated using C-alpha test for unusual distribution of variants between cases and controls, developed by Neale et al. (2011). The C-alpha test is a two-tailed test. This test is integrated in the VAT tools package and also in the GEMINI framework (Paila et al., 2013) for exploring genome variation. VAT tools was used to calculate the C-alpha statistics using 500,000 permutations for all variants with type: “non-synonymous,” “splicing,” “stoploss,” “stopgain,” or “ncRNA.” Only genes with *p* < 0.005 are reported. GEMINI was used to calculate the C-alpha Z-scores (without permutations) using only non-synonymous SNPs. Only genes with *Z*-score < -5 or > 5 are reported.

### Pathway Analysis

Reactome pathways on the genes flagged as significant by the SKAT-O and/or C-alpha statistics was performed using^2^ (Jassal et al., 2020). The tool automatically performs an overrepresentation analysis. The main goal of the pathway analysis in this study is however to group the genes of interest into pathways and explore the possible involvement of these pathways in PXE phenotype modulation. Further, functional data for the significant genes was searched using the Pubmed database. Gene interaction networks were plotted using Genemania and Cytoscape.

### Interleukin B1 Expression

ILB1 expression was evaluated using qPCR in a stimulation experiment, as previously described, in dermal fibroblasts of the patients with a mild or severe phenotype as detailed above and in Table 1 (Lopez-Castejon and Brough, 2011). A detailed description of the methodology of the stimulation experiment can be found in the Supplementary Materials. In brief, at day 1 patient dermal fibroblasts were seeded in triplicate in 60 × 15 mm petri dishes, followed by a 48 h incubation at 37°C and 5% CO_2_. At day 3, medium in all petri dishes was replaced by a 1 μg/mL lipopolysaccharides (LPS, Sigma-Aldrich, Overijse, Belgium) -DMEM solution. After an incubation time of 16 h, the medium in all petri dishes was renewed with a 5 mM ATP (Sigma-Aldrich, Overijse, Belgium) in DMEM solution for 1 h at 37°C/5% CO_2_, followed by RNA extraction with the RNeasy mini kit according to manufacturer’s guidelines (Qiagen, Antwerp, Belgium). Additionally, for each patient, dermal fibroblasts were seeded in triplicate and used as negative control. IL1B expression was evaluated by Quantitative PCR a LightCycler 480 (Roche, Vilvoorde, Belgium) using hypoxanthine phosphoribosyltransferase 1 (*HPRT1*) and tyrosine 3-monooxygenase/tryptophan 5- monooxygenase (*YWHAZ*) as reference genes. Q-PCR data was analyzed using the qBase + software (Biogazelle, Zwijnaarde, Belgium). Differential gene expression was determined using the ΔΔCt method. To quantify statistical significance of differential expression, a 2-sided Mann-Whitney U test was performed (non-parametrical test, not assuming an underlying normal distribution) using the Qbase + software. The significance level was set at 0.05.

**TABLE 1:** Patient characteristics.

**Demographics**			**Phenodex**				***ABCC6* genotype**						**Calcium score**
**Pt**	**M/F**	**Age**	**S**	**E**	**CV**	**GI**	**Pathogenic variant 1**			**Pathogenic variant 2**			
P1	M	40	1	2	2	–	c.1553G > A	p.(R518Q)	C5	del2860_2865	/	C5	6911.4
P2	F	34	1	2	2	–	c.1553G > A	p.(R518Q)	C5	del2860_2865	/	C5	3527.6
P3	M	58	2	2	2	+	c.3490C > T	p.(R1164*)	C5	c.3490C > T	p.(R1164*)	C5	36325.2
P4	F	71	2	2	2	–	c.3421C > T	p.(R1141*)	C5	c.998 + 2	998 + 3delTG	C5	5547.8
P5	F	47	2	2	2	–	c.3032T > C	p.(L1011P)	C3LP	c.3032T > C	p.(L1011P)	C3LP	7234.1
P6	M	66	1	2	1	–	c.3413G > A	p.(R1138Q)	C5	c.2911T > C	p.(W971R)	C3LP	19478
P7	M	59	1	2	1	–	c.3421C > T	p.(R1141*)	C5	c.3421C > T	p.(R1141*)	C5	14.8
P8	F	41	1	1–2	1	–	c.1553G > A	p.(R518Q)	C5	del2860_2865	/	C5	0
P9	F	30	1	1	2	–	c.1321C > T	p.(R441C)	C4	c.3107T > C	p.(F1036S)	C4	6.3
P10	M	55	1	1	1	–	c.3490C > T	p.(R1164*)	C5	c.3490C > T	p.(R1164*)	C5	86.3
P11	F	67	2	2	1	–	c.4198G > A	p.(E1400K)	C5	c.118C > T	p.(P40S)	C3LP	148.2

*The phenodex is a score to describe the severity of the PXE phenotype in a standardized way. Skin phenotype (S): S0, no macroscopic skin lesions; S1* (, papular lesions,; S2 (, =coalesced plaques of papules; Eye phenotype (E): E0 (, no retinopathy,*; E1* (, uncomplicated peau d’orange and/or angioid streaks*; E2, subretinal neovascularization; hemorrhaging and vision loss; Cardiovascular phenotype: CV0* (, no cardiovascular symp*toms; CV1, non-occlusive vasculopathy; CV2, occlusive vessel disease.; Gastrointestinal bleeding (GI): +, yes; -, no. F, female; M, male; Pt, patient code. Pathogenicity classification of the ABCC6 variants was done as previously described (Verschuere et al., 2021): only class 5 pathogenic variants (C5), class 4 likely pathogenic variants (C4) and class 3 likely pathogenic variants (C3LP) were considered as disease-causing variants.*

## Results

### Patient Characteristics

Whole exome sequencing was performed on 11 patients with an extreme cardiovascular PXE phenotype. Six patients had an extremely severe cardiovascular PXE phenotype (P1-P6) and five an extremely mild phenotype (P7-P11). The mean age in the severe group was 52.7 years (range: 34–71) and 50.4 years (range: 30–67) in the mild group. Table 1 gives an overview of the patients characteristics, including age, sex, phenotypic classification (Phenodex), *ABCC6* genotype and Calcium score.

### Variant Calling

GATK yielded 54.606 ± 2.543 variants across the different samples, while Freebayes called 164.904 ± 16.917 variants. None of the variant callers was able to (blindly) call all the variants known to be present in the patient samples (Hosen et al., 2015). After merging the variants called by both methods, the final variant vcf files contained 170.689 ± 14.691 variants. The merged results completely confirmed the snp and small indel results previously reported by Hosen et al. (2015).

### C-alpha and SKAT-O Analyses Identify Enrichment of Genes Involved in Cardiovascular Disease, Inflammation, and Apoptosis

**FIGURE 1:**
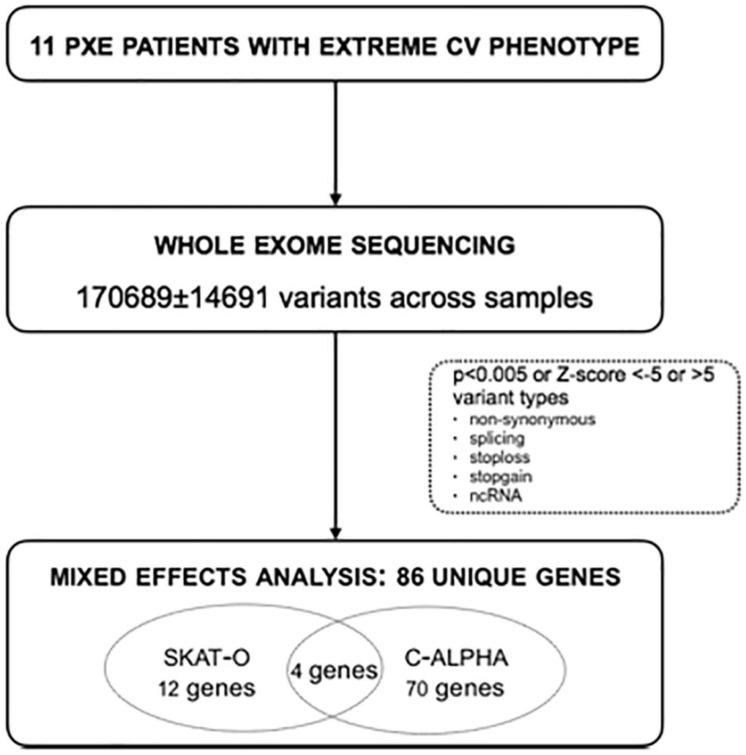
Bioinformatics pipeline. Whole exome sequencing was performed in 11 PXE patients with an extreme cardiovascular (CV) PXE phenotype (severe *n* = 6; mild *n* = 5), based on calcium scoring through computed tomography (CT). Over 170,000 variants were identified, and SKAT-O and C-alpha statistical tests were performed, leading to 16 and 74 potential modifier genes, respectively. In total 86 unique potential modifier genes were identified (no FDR correction). CT, computed tomography; CV, cardiovascular; ncRNA, non-coding ribonucleic acid; SKAT-O, sequence kernel association test–optimal.

The SKAT-O and C-alpha statistics were calculated using all called variants with types “non-synonymous,” “splicing,” “stoploss,” “stopgain,” or “ncRNA,” which yielded, respectively 16 and 74 genes significantly modifying the phenotype severity (Supplementary Tables 1–3). Overall, 86 genes were identified to significantly modify the cardiovascular disease severity in PXE, of which four genes are present in both tests (*HCAR3*, *CNGB3*, *SI*, and *PRKAR1A*) (Figure 1). Forty-six of these genes could be associated with a monogenic phenotype (*n* = 15), a cardiovascular or cerebrovascular phenotype [such as a disease (*n* = 25) or a risk factor (*n* = 22)] or an aberrant mineralization phenotype (*n* = 3; Supplementary Table 4).

Gene interaction analysis demonstrates high network connectivity between most of the 86 identified candidate modifier genes themselves (Supplementary Figure 1) and to a lesser extent with ABCC6 (Supplementary Figure 2). A similar analysis with already known genes associated with human mineralization disorders (summarized in Supplementary Table 5), demonstrates an even denser network connectivity (Supplementary Figure 3).

Reactome pathway analysis revealed that *CDON*, *DLC1*, *DYNC2H1*, *FLG*, *HCAR3*, *IQGAP3*, *OR10AG1*, *OR2L3*, *OR2M*, *OR4A16*, *OR51B4*, *OR5I1*, *OR6C1*, *OR6C70*, *PRKAR1A*, *SI*, and *TLN1* are in signal transduction pathway R-HSA-162582. *NUP210*, *DEGS2*, *MMAB*, *SI*, *AK9*, *PRKAR1A*, and *PLBD1* belong to the metabolism related pathway R-HSA-1430728. *FXYD3*, *TRPV1*, *NUP210*, *RYR3*, and *PRKAR1A* belong to pathway R-HSA-382551 involved in transmembrane transport of small molecules. *NUP210* and *MUC4* belong to the protein metabolism pathway R-HSA-597592.

Table 2 and Supplemental Figure 4 show the processes to which the genes could be linked. Twenty out of the 86 genes have links with cardiovascular disease, 19/86 are linked to apoptosis, 14/86 to inflammation, 5/86 to cell proliferation and 3/86 to calcium homeostasis; for 41/86 genes no relevant data was available.

**TABLE 2:** Potential modifier genes per pathway in which they are involved.

**Pathways**	**Genes**
Cardiovascular disease	ABCA13, AHNAK2, CAMTA2, CDON, **CSF1R**, FLG, HCAR3, MMAB, MYBPHL, **NLRP1**, NLRP11, PDE4DIP, PLBD1, **SELE**, SERPINA9, TOR2A, **TRPV1**, TTN, ULK4, ZNF85
Apoptosis	ACIN1, BRWD1, CAPN14, CDON, CD101, CHAF1A, CHFR, CNGB3, **CSF1R**, DLC1, IGF2-AS, IQGAP3, MUC4, **NLRP1**, NOM1, OR51B4, PRKAR1A, **SELE**, **TRPV1**
Inflammation	AHNAK2, CFH, **CSF1R**, DLC1, HCAR3, HTRA3, MUC4, **NLRP1**, NLRP11, PGLYRP2, PTPN7, **SELE**, TOR2A, **TRPV1**
Calcium homeostasis	PKD1L2, PCLO, RYR3
Cell proliferation	DEGS2, IQGAP3, EPB41L4A, TLN1, GSG2
No relevant data	AK9, ANKRD20A3, APOL5, BZRAP1, CLDN24, C9orf117, DMGDH, DNAH17, DHX57, DYNC2H1, EFCC1, FAM46B, FAM66D, FXYD3, LINC00452, LRRIQ3, MAP1A, NUP210, NWD1, OAF, OR10AG1, OR2L3, OR2M2, OR4A16, OR5I1, OR6C1, OR6C70, OTOP2, PCDHA10, PMS2, PPP1R36, RBM19, RINL, RNU6-28P, SI, SNTG2, SNX18, USP17L7, WIPI1, ZNF28, ZNF417

*Genes involved in two biological processes are underlined; genes involved in three biological processes are in bold.*

### Gene Prioritization Highlights Four Genes Linked by Interleukin 1B Signaling

As apoptotic and inflammatory pathways had been previously suggested to play a role in the PXE pathophysiology and cardiovascular disease is a hallmark of PXE, we focused on these three processes to further prioritize the most interesting candidate modifier genes (Zhong et al., 2016). We found 11 genes that could be associated with at least two of these biological processes and four genes were linked with all three: *NLRP1*, *SELE*, *TRPV1*, and *CSF1R* (Table 2 and Supplementary Figure 4). NLRP1 is activated by proinflammatory signals and forms inflammasomes, which have a role in both inflammation and pyroptosis in multiple organ systems. In the skin NLRP1 induction may lead to skin hyperinflammation and carcinoma (Li et al., 2018) and in the eye, NLRP1 is associated with diabetic retinopathy and acute glaucoma (Chi et al., 2014; Bleda et al., 2016). Further, NLRP1 stimulates the progression of cardiovascular disease, more specifically atherosclerosis and was also linked to peripheral artery disease (Bleda et al., 2014, 2017; Shen et al., 2016). SELE is located downstream of inflammasomes and is activated by inflammatory signals (Milstone et al., 2000; Ley, 2003; Galkina and Ley, 2007). SELE also stimulates atherosclerosis and has both pro- and anti-apoptotic properties (Winkler et al., 2004; Porquet et al., 2011; Signorelli et al., 2012). Furthermore, it is also elevated in patients with peripheral artery disease (Wang and Wang, 2013). For TRPV1 a dual role was described in the context of inflammation, where both pro- and anti-inflammatory effects were observed (Musumeci et al., 2011; Wang et al., 2017). In the context of cardiovascular disease, TRPV1 is rather protective against atherosclerotic plaque formation by reducing lipid storage and diminishing endothelial cell inflammation but also has proapoptotic characteristics (Sappington et al., 2009; Ma et al., 2011; Zwicker et al., 2015; Bleda et al., 2017). Finally, CSF1R-associated signaling can also induce and reduce inflammation, can stimulate atherosclerosis and has anti-apoptotic characteristics (Kovarova et al., 2012; Nandi et al., 2012; Wei et al., 2015).

**FIGURE 2:**
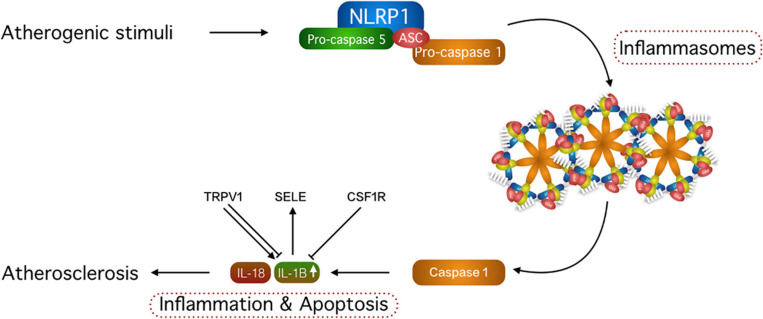
Identification of a common denominator, IL1B. NLRP1, TRPV1, SELE, and CSF1R can all signal through IL1B. NLRP1 signals through inflammasomes leading to an upregulation of IL1B and its downstream effects, while CSF1R has an inhibitory effect on the cytokine. TRPV1 can both stimulate and inhibit IL1B. SELE is located downstream of and is stimulated by IL1B. ASC, adaptor protein; IL-18, interleukin 18; IL-1B, interleukin 1B.

Interestingly, NLRP1, SELE, TRPV1, and CSF1R can all signal through the cytokine interleukin 1B, which is linked with cardiovascular disease, apoptosis and inflammation (Ley, 2003; Musumeci et al., 2011; Wei et al., 2015; Chowdhury et al., 2017; Wang et al., 2017; Figure 2). We were therefore interested to see whether there was differential expression of IL1B in patients with a mild cardiovascular phenotype compared to those with a severe cardiovascular phenotype. For this, we performed a preliminary IL1B stimulation experiment in dermal fibroblasts of patients with an extremely mild and severe cardiovascular phenotype and healthy controls, which showed a significant upregulation of IL1B in severely affected PXE patients compared to mildly affected patients (eightfold; *p* < 0.001, 95%CI: 2.9–24.6) and to healthy controls (51-fold; *p* < 0.001, 95%CI: 24.2–107.2; Supplementary Figure 5).

## Discussion

PXE is a genetic disorder for which nearly no genotype-phenotype correlations exist between the causal gene *ABCC6* and the eye, skin and cardiovascular features. Therefore, other mechanisms must play a role in explaining the extensive disease variability between patients. In this study we used an extreme phenotypes approach using whole exome sequencing to identify new candidate modifier genes for the cardiovascular phenotype of PXE.

Using mixture of effects analyses (C-alpha and SKAT-O) we found 86 genes to be associated with a severe cardiovascular PXE phenotype. Taking into account the risk of false positive results (type I error), we used two strategies to identify potential modifier genes for the PXE cardiovascular phenotype. First, we focused on those genes with a significant result in both the C-alpha and SKAT-O test: *HCAR3*, *CNGB3*, *SI*, and *PRKAR1A*. HCAR3 is a G-protein-coupled receptor, mediating antilipolytic effects, and is also abundantly present in neutrophils, where it is involved in the chemotactic response (Ahmed et al., 2009; Irukayama-Tomobe et al., 2009). Thus, it could have a protective role in cardiovascular disease, however no extensive functional data is present. For *PRKAR1A*, *SI* and *CNGB3* no relevant associations with cardiovascular disease were identified, except for a relatively weak association between *CNGB3* and varicosity (Fukaya et al., 2018). Interestingly, *PRKAR1A* was shown to directly influence *RUNX2* expression, a master regulator of osteogenesis which is known to be upregulated in PXE (Hosen et al., 2014; Zhang et al., 2014). It will therefore be of interest to validate *PRKAR1A* further as a potential modifier for ectopic mineralization.

Second, we looked deeper into the (functional) data already known for all genes with an association with the severity of the cardiovascular PXE phenotype. Interestingly, several have been suggested as biomarker, susceptibility or modifier gene for cardiovascular phenotypes associated with vessel calcification (such as coronary artery disease, ischemic stroke, athero, and arteriosclerosis), risk factors (hypertension, obesity, diabetes mellitus, cholesterol metabolism) and disease mechanisms which may worsen cardiovascular disease and calcification (inflammation, ischemia/hypoxia and endothelial dysfunction). Indeed, besides being a common feature of stroke and atherosclerosis, arterial calcification shares many risk factors including diabetes, dyslipidemia, hypertension and inflammation (Wu et al., 2017; Chellan et al., 2018). Recent studies have also indicated that endothelial cells participate in vascular calcification via endothelial-mesenchymal transition, cytokine secretion, extracellular vesicle synthesis, angiogenesis regulation and hemodynamics (Yuan et al., 2021).

Besides associations with bone disorders with disturbed mineralization (osteoporosis and osteopetrosis) for the *CSF1R* and *TLN1* genes, an interesting observation was done in an altered MyBP-C murine model, in which the cardiac myosin heavy chain and titin-binding domain were replaced by other amino acids. In these mice a phenotype occurs, showing a.o. dystrophic cardiac calcification (McConnell et al., 1999). Dystrophic cardiac calcification has been attributed to Abcc6 deficiency in the so-called dyscalc (DCC) mouse model (Meng et al., 2007). Though *TTN* is an extremely polymorphic gene and interpretation of its genetic variation can be challenging, this murine link with titin makes further investigation of *TTN* variants as modifiers of calcification worthwhile.

We also explored in which pathways the genes in the SKAT-O/C-alpha significant gene list are present. Several genes are in signal transduction pathway R-HSA-162582 and most of them (*FLG*, *HCAR3*, *OR10AG1*, *OR2L3*, *OR2M2*, *OR4A16*, *OR51B4*, *OR5I1*, *OR6C1*, *OR6C70*, *PRKAR1A*, *SI*, and *TLN1)* are involved in signaling by G protein-coupled receptors (GPCR). The human genome encodes thousands of G protein-coupled receptors (Vassilatis et al., 2003). Several hundred detect hormones, growth factors, and other endogenous ligands. Growth factors are known to modulate the PXE phenotype. The VEGF (vascular endothelial growth factor gene) is modulating the ocular symptoms resulting from PXE (Zarbock et al., 2009; De Vilder et al., 2020). Activation of the MAP kinase signal transduction and the ERK1/2 cascade leads to the significant inhibition of the expression of ABCC6 in HepG2 and Caco-2 cell lines (Varadi et al., 2011). *FLG*, *SI*, *TLN1* are involved in the VEGF receptor signal transduction and MAP kinase signaling. More generally, *FLG*, *KSR2*, *PRKAR1A*, *SI*, and *TLN1* are involved signal transduction of several growth factor signals and MAP kinase regulation.

*NUP210*, *DEGS2*, *MMAB*, *SI*, *AK9*, *PRKAR1A*, and *PLBD1* belong, together with ENPP1 to the metabolism related pathway R-HSA-1430728. As *ENPP1* is a known disease-causing gene of PXE [4], the other genes in this pathway might also act as possible modifiers. A group of genes that stand out are *FXYD3*, *TRPV1*, *NUP210*, *RYR3*, and *PRKAR1A* which are involved in transmembrane transport of small molecules. *ABCC6* also belongs to this pathway (R-HSA-382551) and it is thus plausible that other genes in the same pathway might modify the PXE phenotype. *NUP210* and *MUC4* belong together with *GGCX* and *VKORC1* to the protein metabolism pathway R-HSA-597592. As these enzymes of the vitamin K-cycle have been demonstrated to be involved in PXE and the PXE-like syndrome with coagulation factor deficiency, the other genes in this reactome pathway are PXE modifier candidates (Li et al., 2009; Vanakker et al., 2010).

It is important to remember that our approach is based on identifying candidate modifier genes based on a cumulative effect of rare variants. It can therefore be assumed that several of these pathways and mechanisms are simultaneously at play to influence disease severity. Whether some of these genes are more important than others in the specific context of PXE and the exact mechanisms underlying these interactions remain to be elucidated.

Due to the important role of PPi in the pathophysiology of PXE, it may be surprising that in our analysis no genes surfaced which are directly involved in the PPi metabolism. The same is true for the traditional genes which are involved in mineralization homeostasis and prevention of ectopic mineralization, such as *MGP* or *AHSG*. We cannot exclude that variants in these genes were missed due to the small and very selected patient population in this study, but may still be present in other patients. The results of this study are a first suggestion for candidate modifier genes and by no means should the list of identified genes be seen as exhaustive. While it remains difficult to estimate the definite number of modifier genes that would be relevant in an individual PXE patient, it is likely that these modifier genes are not identical in all patients. On the other hand the goal of this study is to identify genes with a high variant burden, with each variant having a relatively small individual effect. We might assume that functional variants in genes that are directly related to PPi metabolism and mineralization will have a larger individual effect; hence the variant burden can be relatively low which would explain why these genes do not end up among the significant results. While it will be interesting to also study the role of sequence variants in these genes more in detail, our results suggest that the identification of modifier genes cannot be limited to these “obvious” candidate genes.

We found that many of the candidate modifier genes could be linked with cardiovascular disease, inflammation and apoptosis. As inflammation and apoptosis have been previously implicated in the PXE pathophysiology and cardiovascular disease is part of the classic PXE triad, this confirms the validity of our results (Stoneman and Bennett, 2004; Van Vre et al., 2012; Zhong et al., 2016; Wu et al., 2017). Further prioritization based on these three processes led to four candidate modifier genes of the severity of the cardiovascular PXE phenotype: *NLRP1*, *SELE*, *CSF1R*, and *TRPV1*. Interestingly, these four genes share a common denominator as they can all signal through IL1B, a member of the IL1 family, which also consists of IL1alpha and IL1-ra, the latter being an endogenous IL1 inhibitor. IL1 signaling, both through direct contact (IL1alpha) and at a distance (IL1B), is implicated in a number of cardiovascular diseases, including atherosclerosis. It induces inflammatory functions of human endothelial cells, stimulates vascular smooth muscle cells—important in atherogenesis—through autocrine production of platelet-derived growth factor and leads to IL6 activation which in turn activates other atherothrombotic mediators, such as fibrinogen, plasminogen activator inhibitor and C-reactive protein (CRP) (Libby, 2017). Interestingly, IL6 upregulation was identified in peripheral artery disease (Wang et al., 2017). In addition, IL1 also alters cardiac myocyte functioning, thus impairing the contractile function of the heart. Moreover, IL1B can aggravate ischemia-reperfusion injury and cardiac remodeling after experimental myocardial infarction (Libby, 2017). Interestingly, a worse cardiac outcome after experimental ischemia-reperfusion has been shown in the PXE mouse model (Mungrue et al., 2011).

The significantly upregulated activation of the inflammatory response IL1B in cells of severely affected PXE patients compared to both mildly affected patients and healthy controls, with respective fold changes of 8 and 51 (Supplementary Figure 5), may point toward a role for IL1B in the severity of the cardiovascular PXE phenotype though further experimental validation will be needed to make a more robust conclusion. While *TRPV1 and* CSF1R have been demonstrated to activate the NLRP3 inflammasome in astrocytes and microglia cells, it has also been demonstrated that various types of crystals—cholesterol crystals but also calcium phosphate crystals such as hydroxyapatite, which is the relevant crystal type in PXE—can activate inflammasomes through lysosomal rupture and subsequent cathepsin B release (Usui et al., 2012; Yang et al., 2019; Hagan et al., 2020). Though most reports are on the NLRP3 and not the NLRP1 inflammasome, it cannot be excluded that a similar mechanism exists. As such the genomic variants we identified may very well not be the cause of IL1B increase, which can be directly due to the ectopic hydroxyapatite crystals in PXE. While this could explain while IL1B expression levels are higher in PXE cells compared to controls without stimulation, we would expect that the expression levels in this baseline condition would be higher in the severe PXE group compared to the mild group as they have much more calcification, which is not the case. Nonetheless, the PXE-induced inflammasome activation suggests that involved genes may influence the severity of cardiovascular disease like IL1B has been shown to contribute to the progression of atherosclerosis (Kirii et al., 2003; Chi et al., 2004). The involvement of IL1B may also be interesting from a therapeutic point of view, as anti-IL1B agents were shown to significantly decrease the risk of a new cardiovascular event in myocardial infarction patients in a large RCT (CANTOS study; Ridker et al., 2011).

Regarding the limitations of this study, it is important to note that with the use of the SKAT-O and C-alpha tests rare variants with a minimally positive, negative or neutral effect are all included and a combined effect is predicted, not taking into account whether the effect is aggravating or protective. These tests will also not detect any differences in expression, e.g., due to epigenetic changes, in potential modifier genes. A second limitation is the small sample size that was used, which is a known problem in studying rare diseases. To overcome this, we used extreme phenotype sampling and specific statistical tests to increase power to be able to draw conclusions from a relatively small cohort. Nonetheless, a validation of the results—in particular the prioritized candidate modifier genes—in larger, independent cohorts of PXE patients as well as experimental validation of the effects of the candidate modifier genes using e.g., genome editing technologies in PXE cell and animal models is essential before they can be implemented in clinical practice. A limitation to study the role of IL1B is the use of fibroblasts, which can be explained by the inavailability of smooth muscle vascular cells from the investigated patients. To make definite conclusions concerning the role of IL1B in PXE, there should be a validation of the expression results in a larger cohort as well as in smooth muscle vascular cells.

In conclusion, this preliminary study explored for the first time the cumulative effect of rare variants on the severity of cardiovascular disease in PXE. Using whole exome sequencing of extreme phenotypes and mixture of effects analyses, SKAT-O and C-alpha, a panel of 86 novel candidate modifier genes were identified, among which four candidate genes, i.e., *NLRP1*, *SELE*, *CSF1R*, and *TRPV1*, could be prioritized for the cardiovascular phenotype of PXE. Though further validation of the suggested modifier genes and pathways in independent and larger patient cohorts is essential as well as wet-lab validation of their putative effects, this panel can aid in risk stratification and genetic counseling of PXE patients and will help to gain new insights in the PXE pathophysiology.

## Data Availability Statement

The datasets for this article are not publicly available because of GDPR regulations. Requests to access the datasets should be directed to OV, olivier.vanakker@ugent.be.

## Ethics Statement

The studies involving human participants were reviewed and approved by the Ghent University Hospital Ethics Committee. The patients/participants provided their written informed consent to participate in this study.

## Author Contributions

OV and FV conceptualization of bioinformatics study design and approach was done. FV bioinformatics pipeline, including SKAT-O and C-alpha tests, was performed. ED and FV data interpretation of results from bioinformatics pipeline and the manuscript was written. ED study design and experimental setup for functional validation and performed all wet lab experiments and data analysis for the IL1B stimulation experiments. GL and LM performed Agatston scoring for French patients and provided necessary fibroblast cultures. OV supervised the writing process and reviewed the manuscript at different stages. PC supervised the laboratory processes. All authors contributed to the article and approved the submitted version.

## Conflict of Interest

The authors declare that the research was conducted in the absence of any commercial or financial relationships that could be construed as a potential conflict of interest.

## Footnotes

1 http://picard.sourceforge.net

2 http://www.reactome.org/PathwayBrowser/
