# Supplementary material for: Rare Modifier Variants Alter the Severity of Cardiovascular Disease in Pseudoxanthoma Elasticum: Identification of Novel Candidate Modifier Genes and Disease Pathways Through Mixture of Effects Analysis
Source: Front Cell Dev Biol. 2021 Jun 8;9:612581. doi: 10.3389/fcell.2021.612581 (PMC8218811; doi:10.3389/fcell.2021.612581)
Supplement: Supplementary file 1 [file Data_Sheet_1.PDF]

Supplementary Data

Supplementary Table 1. SKAT-O test: significant genes.

Refgene name	Sample size	Q_stats	p-value
BRWD1	10	NAN	0.0020444
OR51B4	10	NAN	0.0020444
TOR2A	11	4.31597E- 33	0.00264177
ULK4	11	4.31597E- 33	0.00265131
HCAR3	11	NAN	0.00312239
SI	11	2.8693E-37	0.0032738
CNGB3	11	NAN	0.0032859
WIPI1	11	1.32714E-24	0.00344425
TLN1	10	242.77556E- 61	0.00351608
PRKAR1A	11	NAN	0.00355445
PPP1R36	8	7.09073E-30	0.00359424
CHFR	9	1.74651E-18	0.00366736
C9orf117	10	2.8693E-37	0.00438343
IGF2-AS	7	7.09073E-30	0.00468057
LRRIQ3	10	2.77556E-61	0.00470279
CAMTA2	10	2.77556E- 61	0.00496194

This table gives an overview of the genes associated with severe cardiovascular disease in PXE.

Supplementary Table 2. C-alpha test: significant genes.

refgene name	sample_size_Calpha	num_variants_Calpha	total_mac_Calpha	statistic_Calpha	pvalue_Calpha	std_error_Calpha	#permutations
BZRAP1	9	2	8	4.59968	0.00380399	0.95895	500000
PCLO	11	5	25	2.48999	0.00429999	0.764644	500000
DEGS2	9	2	8	2.40535	0.00570399	0.660998	500000
PCDHA10	11	5	12	4.15349	0.00748399	1.54263	500000
EFCC1	9	3	7	2.06158	0.00785398	0.79707	500000
PMS2	11	4	14	3.98062	0.00838598	1.11698	500000
PLBD1	11	3	13	0.761798	0.00843198	0.437635	500000
DMGDH	11	3	16	2.60352	0.010756	0.85527	500000
HCAR3	11	3	17	1.63225	0.01182	0.633077	500000
MMAB	10	2	14	0.0126229	0.012012	0.287518	500000
MAP1A	7	2	6	1.68585	0.01433	0.675796	500000
RNU6-28P	8	5	12	1.89737	0.014372	0.939245	500000
RINL	7	3	6	1.13951	0.014416	0.54516	500000
DYNC2H1	11	4	12	1.63841	0.016072	0.863739	500000
USP17L7	10	5	27	1.27961	0.016564	0.651244	500000
SNX18	10	2	8	0.704334	0.01672	0.320828	500000
FAM66D	10	10	44	2.05981	0.019178	1.01052	500000
CFH	11	2	8	1.10187	0.019312	0.532876	500000
PRKAR1A	11	3	18	0.432672	0.019622	0.446481	500000
AK9	9	3	15	0.431845	0.01978	0.397409	500000
DLC1	11	2	10	0.655462	0.020402	0.460217	500000
CLDN24	11	2	7	1.56715	0.020692	0.752003	500000
CNGB3	11	2	10	0.991684	0.024694	0.528475	500000
NUP210	11	4	26	1.22127	0.0278819	0.797123	500000
DNAH17	11	7	25	0.94894	0.0280899	0.66486	500000
GSG2	11	2	6	3.17115	0.0297299	1.23497	500000
NOM1	11	5	12	4.12373	0.0299059	1.61289	500000
DHX57	11	2	9	0.980053	0.0299679	0.569177	500000
ANKRD20A3	11	3	17	0.373721	0.0302099	0.4062	500000
SI	11	2	6	3.17115	0.0302479	1.23417	500000
RBM19	11	2	4	2.24234	0.0305219	0.874241	500000
CSF1R	11	2	9	1.23879	0.0308879	0.666026	500000
FXYD3	10	2	10	0.894427	0.0313179	0.568655	500000
OAF	11	2	9	1.23879	0.0313499	0.667735	500000
SERPINA9	11	5	23	1.68961	0.0326959	1.01881	500000
CAPN14	6	3	12	1.35873	0.0331359	0.629916	500000
ZNF417	6	3	10	0.961524	0.0338739	0.59267	500000
OR6C70	11	2	7	1.3235	0.0348479	0.751787	500000
SNTG2	9	2	7	1.59111	0.0359419	0.787273	500000
CD101	11	2	8	0.690066	0.0372979	0.403726	500000
ZNF28	11	2	7	2.29812	0.0380879	1.00874	500000
TRPV1	11	2	5	1.89989	0.0383899	0.75893	500000
IQGAP3	11	2	6	1.30776	0.0407199	0.814231	500000
RYR3	11	2	6	1.30776	0.0410559	0.814479	500000
MYBPHL	11	2	7	1.3235	0.0410959	0.861902	500000
OR6C1	11	4	15	2.89732	0.0415379	1.3154	500000
PTPN7	7	2	7	0.881774	0.0422919	0.559024	500000
LINC00452	10	2	5	2	0.0432439	0.923333	500000
NWD1	11	3	14	0.388305	0.0433379	0.431997	500000
CHAF1A	11	3	6	1.38722	0.0437719	0.748887	500000
NLRP11	11	2	6	1.38722	0.0441459	0.749132	500000
ACIN1	11	2	10	1.56943	0.0468879	0.901118	500000
APOL5	11	2	12	1.0997	0.0473059	0.736991	500000
EPB41L4A	11	4	20	2.21952	0.0473259	1.27994	500000
OR2M2	11	2	10	1.56943	0.0473399	0.903885	500000
CDON	10	4	14	1.04257	0.0476299	0.69359	500000
HTRA3	5	3	8	1.23091	0.0491299	0.866757	500000

This table gives an overview of the genes associated with severe cardiovascular disease in PXE.

Supplementary Table 3. C-alpha test: significant genes with the GEMINI workflow.

Gene	T	c	Z
OTP2	-2.22044604925e-16	4.94433440339	-9.98587918667e- 17
AHNAK2	-138.280991736	705.070691893	5.20770235606
PDE4DIP	-125.867768595	607.030257496	5.10868780485
OR5I1	10.2479338843	4.2005327505	5.00016259898
SELE	12.2314049587	5.92172665802	5.02634352
FLG	47.5454545455	74.1650160508	5.52089460162
PGLYRP2	12.0	4.70049859982	5.53489367117
FAM46B	12.8429752066	5.17792500512	5.64400989062
PKD1L2	111.611570248	381.43501127	5.71477243635
ABCA13	79.0	169.693326959	6.06449593227
OR2L3	17.0578512397	6.90116795301	6.49326345598
OR10AG1	21.2561983471	9.61409739772	6.85537687513
ZNF85	22.5619834711	9.12027866949	7.47090514764
NLRP1	59.8925619835	44.0892015573	9.02000491163
TTN	251.099173554	669.101837306	9.70731610646
MUC4	569.504132231	1603.35154703	14.222714818

This table gives an overview of the genes associated with severe cardiovascular disease in PXE.

Supplementary Table 4. Identified candidate modifier genes with a known associated monogenic disease and/or association with cardio- and cerebrovascular disease and risk factors.

Gene	Name	Associated monogenic phenotype(s)	Associations with cardiovascular disease/risk factor	Association type	Pubmed ID
**ABCA13**	ATP binding cassette subfamily A member 13	None	Graft stenosis after CABG	Genetic variation	25881214
**ACIN1**	apoptotic chromatin condensation inducer 1	None	None		
**AHNAK2**	AHNAK nucleoprotein 2	Charcot-Marie-Tooth disease	None		
**AK9**	adenylate kinase 9	None	None		
**BRWD1**	bromodomain and WD repeat domain containing 1	None	Cholesterol and LDL metabolism	Genetic variation	30275531
**CAMTA2**	calmodulin binding transcription activator 2	None	Left ventricular hypertrophy	Genetic variation	16678087, 26886562
**CAPN14**	calpain 14	None	Inflammation	Genetic variation	28131390
**CDON**	cell adhesion associated, oncogene regulated	Holoprosencephaly	None		
**CD101**	CD101 molecule	None	ID Diabetes mellitus	Genetic variation<br>Biomarker	27888582<br>31199784
**CFH**	complement factor H	Basal laminar drusen<br>Complement factor H deficiency	Cerebrovascular disease<br>Hypertension<br>Coronary artery disease	Biomarker<br>Altered expression<br>Genetic variation<br>Altered expression<br>Genetic variation	21695352<br>21695352<br>25402348<br>31296141<br>20127520
**CHAF1A**	chromatin assembly factor 1 subunit A	None	Diabetes mellitus<br>Obesity	Biomarker<br>Genetic variation	29121716<br>28132653
**CHFR**	checkpoint with forkhead and ring finger domains	None	Body Mass Index	Genetic variation	30595370
**CNGB3**	cyclic nucleotide gated channel subunit beta 3	Achromatopsia 3	Varicosity	Genetic variation	30566020
**CSF1R**	colony stimulating factor 1 receptor	Brain abnormalities, neurodegeneration and dysosteosclerosis<br>Leukoencephalopathy, diffuse hereditary, with spheroids	Osteopetrosis	Biomarker	28383543
**DEGS2**	delta 4-desaturase, sphingolipid 2	None	Sudden cardiac arrest<br>Diabetes mellitus	Genetic variation<br>Biomarker	21658281<br>29319171
**DLC1**	DLC1 Rho GTPase activating protein	None	Venous thromboembolism<br>Arteriosclerosis	Genetic variation<br>Altered expression	23509962<br>30231995
**DMGDH**	dymethylglycine dehydrogenase	Dimethylglycine dehydrogenase deficiency	None		
**DYNC2H1**	dynein, cytoplasmic 2, heavy chain 1	Short-rib thoracic dysplasia 3 with or without polydactyly	Body Mass Index	Genetic variation	30595370
**EPB41L4A**	erythrocyte membrane protein band 4.1 like 4A	None	None		
**FLG**	fillagrin	Ichthyosis vulgaris	Cholesterol, LDL and HDL metabolism	Genetic variation	23460889
**HCAR3**	hydroxycarboxylic acid receptor 3	None	Atherosclerosis	Biomarker	30270326
**IGF2-AS**	IGF2 antisense RNA	None	ID diabetes mellitus<br>Systolic pressure	Genetic variation<br>Genetic variation	26301688<br>30595370
**IQGAP3**	IQ motif containing GTPase activating protein 3	None	None		
**MAP1A**	microtubule associated protein 1A	None	Lipid metabolism	Genetic variation	30926973
**MMAB**	metabolism of cobalamin associated B	Methylmalonic aciduria, vitamin B12-responsive, cblB type	Cholesterol and HDL metabolism	Genetic variation	29507422

**MUC4**	mucin 4, cell surface associated	None	Familial cardiomyopathy	Altered expression	29281999
**MYBPHL**	myosin binding protein H like	None	Cholesterol and LDL metabolism	Genetic variation	23063622
**NLRP1**	NLR family pyrin domain containing 1	Autoinflammation with arthritis and dyskeratosis<br>Palmoplantar carcinoma, multiple self-healing	Cholesterol metabolism	Genetic variation	23063622
**NOM1**	nucleolar protein with MIF4G domain 1	None	None		
**NUP210**	nucleoporin 210	None	Coronary artery disease<br>ID Diabetes mellitus<br>Body Mass Index	Genetic variation<br>Genetic variation<br>Genetic variation	29695241<br>29695241<br>30595370
**PCLO**	piccolo presynaptic cytomatrix protein	Pontocerebellar hypoplasia, type 3	NID Diabetes mellitis<br>Hypoxia-ischemia brain	Genetic variation<br>Biomarker	18647954<br>31580838
**PDE4DIP**	phosphodiesterase 4D interacting protein	None	Ischemic stroke	Genetic variation	25961151
**PMS2**	PMS1 homolog 2	Colorectal cancer, hereditary nonpolyposis, type 4<br>Mismatch repair cancer syndrome 4	None		
**PRKAR1A**	protein kinase cAMP-dependent type I regulatory subunit alpha	Acrodysostosis 1, with or without hormone resistance Carney complex, type 1<br>Myxoma, intracardiac<br>Pigmented nodular adrenocortical disease, primary, 1	None		
**RYR3**	ryanodine receptor 3	None	Atherosclerosis<br><br>Hypertension<br>Ischemic stroke	Genetic variation<br>Biomarker<br>Genetic variation<br>Genetic variation	24561552<br>20009918<br>29590321<br>24423397
**SELE**	selectin E	None	Atherosclerosis<br>Premature coronary artery disease	Genetic variation<br>Genetic variation	20818642<br>29288721
**SERPINA9**	serpin family A member 9	None	Ischemic stroke<br>Coronary heart disease	Genetic variation<br>Genetic variation	18799872<br>18799872
**SI**	sucrase-isomaltase	Sucrase-isomaltase deficiency, congenital	NID Diabetes mellitus	Biomarker	31553294
**SNTG2**	syntrophin gamma 2	None	Body Mass Index	Genetic variation	30595370
**TLN1**	talin 1	None	Osteoporosis<br>Coronary artery dissection<br>Carotid stenosis<br>Cardiomyopathy	Biomarker<br>Genetic variation<br>Biomarker<br>Altered expression	18924182<br>30888838<br>31215474<br>28698364
**TOR2A**	torsin family 2 member A	None	Atherosclerosis/arteriosclerosis<br>Hypertension<br>Cardiovascular disease<br>Familial cardiomyopathy<br>Endothelial dysfunction	Biomarker<br>Biomarker<br>Biomarker<br>Biomarker<br>Biomarker	31584433<br>30581101<br>30581101<br>28333148<br>28647373
**TRPV1**	transient receptor potential cation channel subfamily V member 1	None	Atherosclerosis<br>Obesity	Biomarker<br>Biomarker	21908651, 29335450<br>30496795, 28104916
**TTN**	titin	Cardiomyopathy, dilated, 1G<br>Cardiomyopathy, familial hypertrophic, 9<br>Muscular dystrophy, limb-girdle, autosomal recessive 10 Myopathy, myofibrillar, 9, with early respiratory failure Salih myopathy<br>Tibial muscular dystrophy, tardive	Sudden cardiac death<br>Myocardial infarction<br>Dystrophic calcification	Genetic variation<br>Biomarker<br>Biomarker	12221049<br>12221049<br>12221049
**ULK4**	unc-51 like kinase 4	None	Hypertension<br><br>Coronary heart disease	Biomarker<br>Genetic variation<br>Genetic variation	19430479<br>28498854<br>21347282
**WIPI1**	WD repeat domain, phosphoinositide interacting 1	None	Right ventricular failure	Biomarker	31021818
**ZNF85**	zinc finger protein 85	None	Thrombocytopenia	Genetic variation	16304054

This table gives an overview of the identified candidate modifier genes which are associated with a monogenic phenotype. Also associations with cardiovascular disease and/or risk factors are listed. The description of the association type is based on the DisGeNET platform (www.disgenet.org). Biomarker: This relationship indicates that the gene/protein either plays a role in the etiology of the disease (e.g. participates in the molecular mechanism that leads to disease) or is a biomarker for a disease. Genetic variation: This relationship indicates that a sequence variation (a mutation, a SNP) is associated with the disease phenotype, but there is still no evidence to say that the variation causes the disease. Altered expression: This relationship indicates that an altered expression of the gene is associated with the disease phenotype. CABG: coronary artery bypass graft; ID: insulin-dependent; NID: non-insulin-dependent

Supplementary Table 5. Known genes involved in human ectopic mineralization phenotypes

Gene	Associated mineralization phenotype
ABCC6	Pseudoxanthoma elasticum
ADIPOQ	Adiponectin deficiency
ANK	Craniometaphyseal dysplasia
APOE	Atherosclerosis
ATF4	Atherosclerosis
CAR2	Carbonic anhydrase II deficiency
CASR	Familial hypocalciuric hypercalcaemia type 1
ENPP1	Generalized arterial calcification of infancy
FAM20A	Enamel-renal syndrome
FGF23	Hereditary hyperphosphataemic tumoral calcinosis
GALNT3	Hereditary hyperphosphataemic tumoral calcinosis
GGCX	PXE-like syndrome with coagulopathy
KL	Hereditary hyperphosphataemic tumoral calcinosis
MGP	Keutel syndrome
NT5E	Calcification of joints and arteries
SAMD9	Hereditary normophosphataemic tumoral calcinosis
SLC20A2	Primary familial basal ganglia calcifications
SPP1	Athersclerosis
TNFRSF11B	Hypertrichosis universalis congenita

Supplementary Figure 1. Gene interaction analysis of the 86 identified candidate modifier genes.

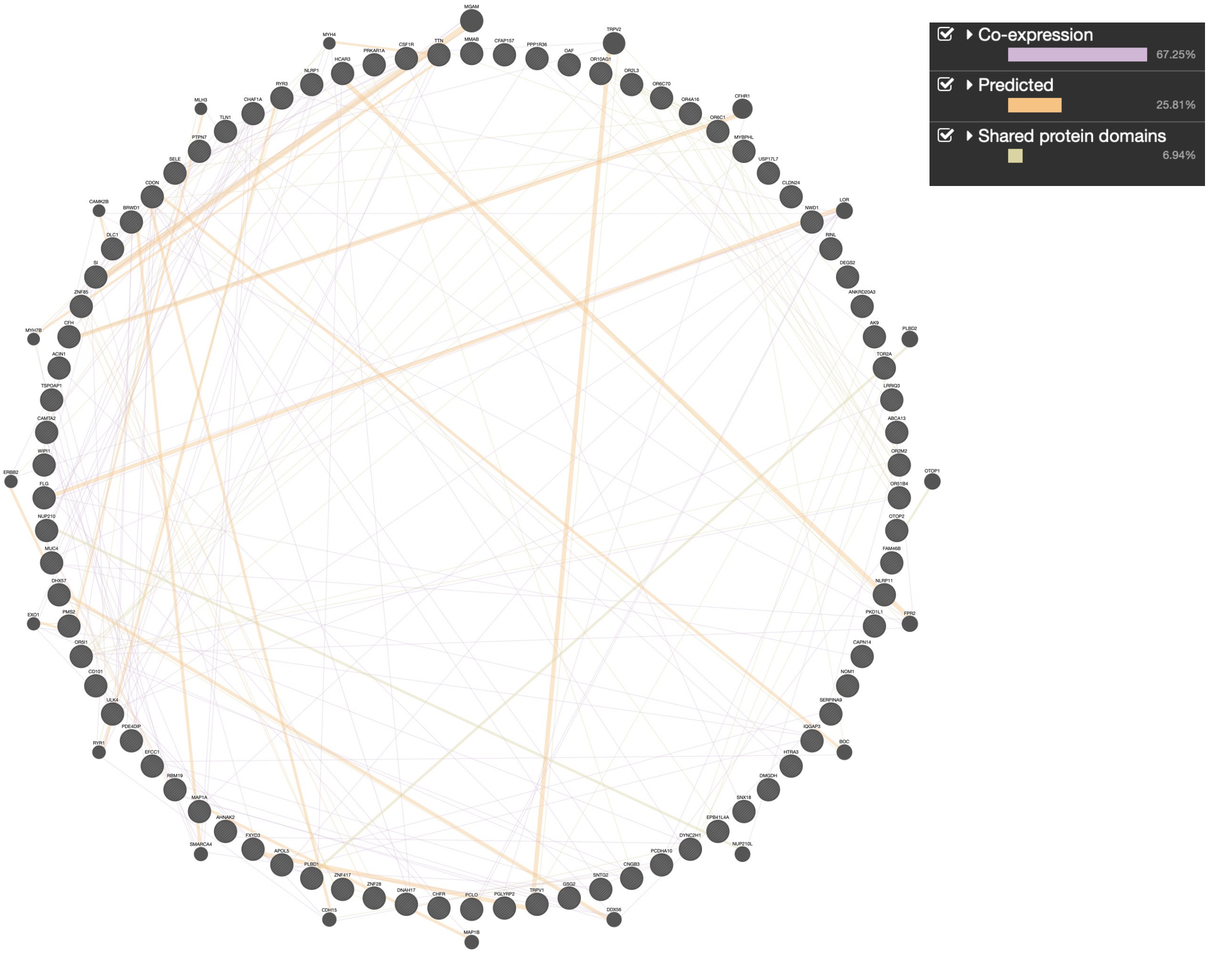

Supplementary Figure 2. Gene interaction analysis of the 86 identified candidate modifier genes and ABCC6 (marked in red).

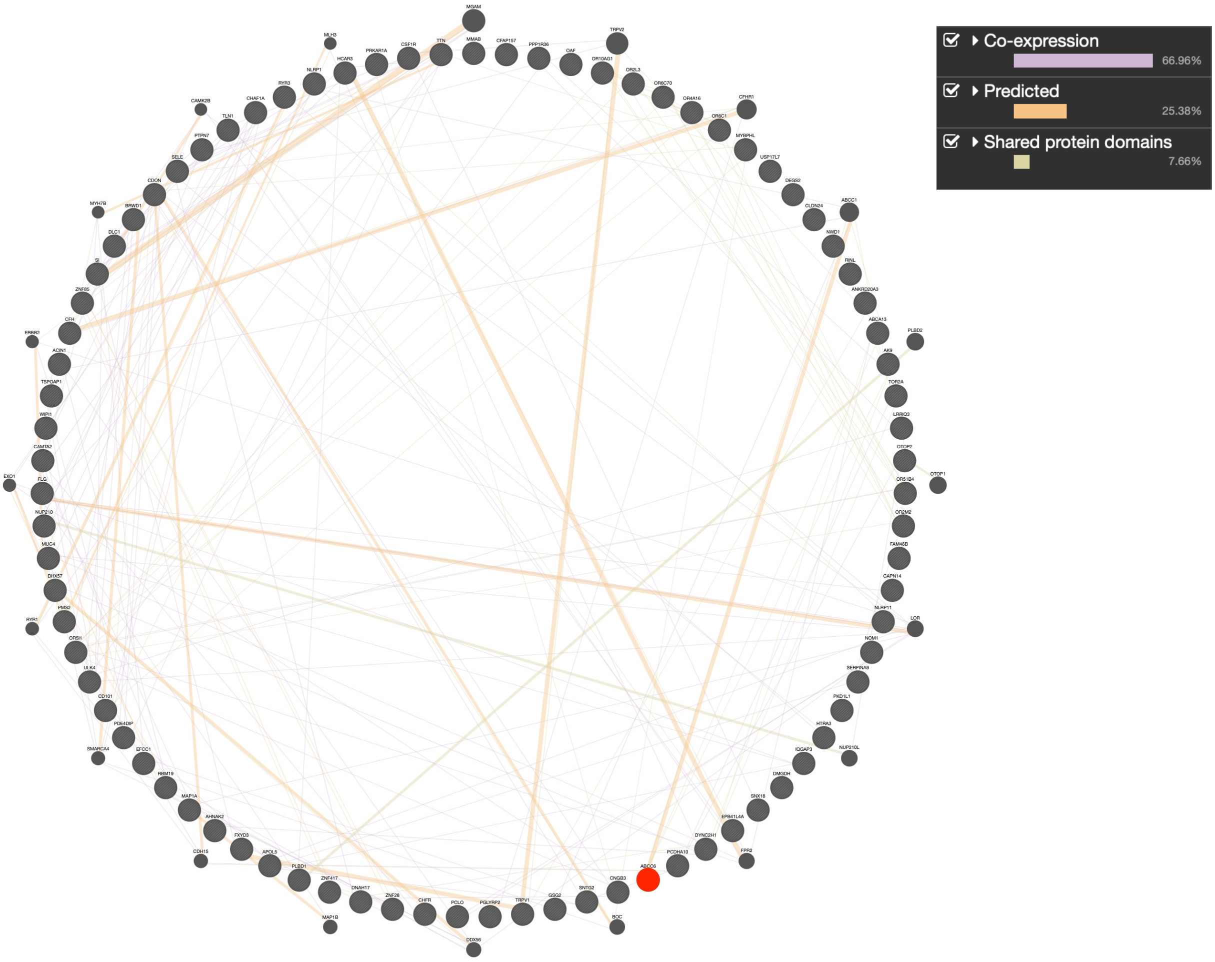

Supplementary Figure 3. Gene interaction analysis of the 86 identified candidate modifier genes and known genes for human ectopic mineralization disorders.

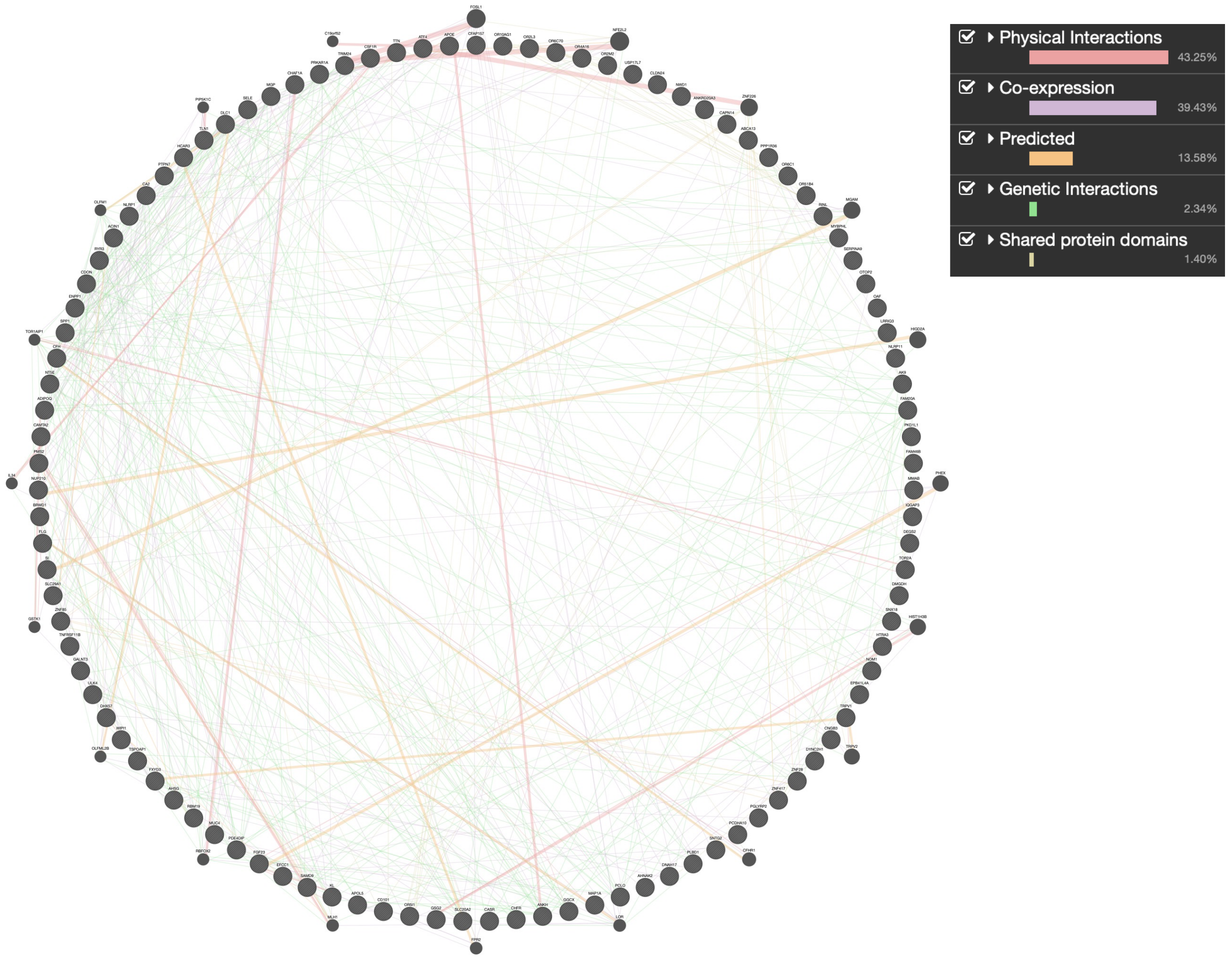

Supplementary Figure 4. Biological processes to which the 86 genes from the C-alpha and SKAT-O test can be linked.

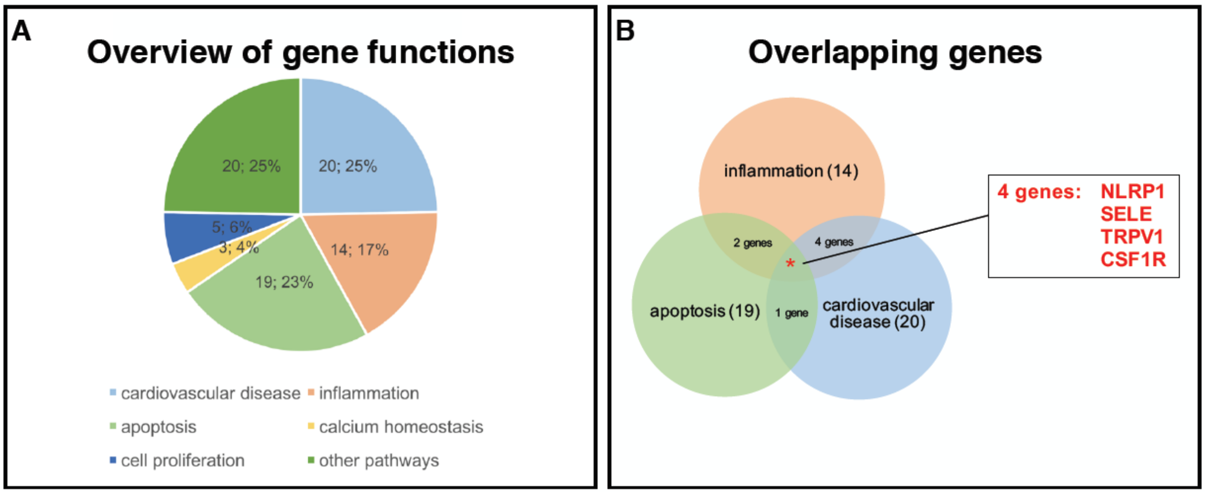

Twenty out of the 86 genes have links with cardiovascular disease, 19/86 are linked to apoptosis, 14/86 to inflammation, 5/86 to cell proliferation and 3/86 to calcium homeostasis; for 41/86 genes no relevant data was available.

Supplementary Figure 5. IL1B expression in healthy controls and PXE patients: baseline and after stimulation.

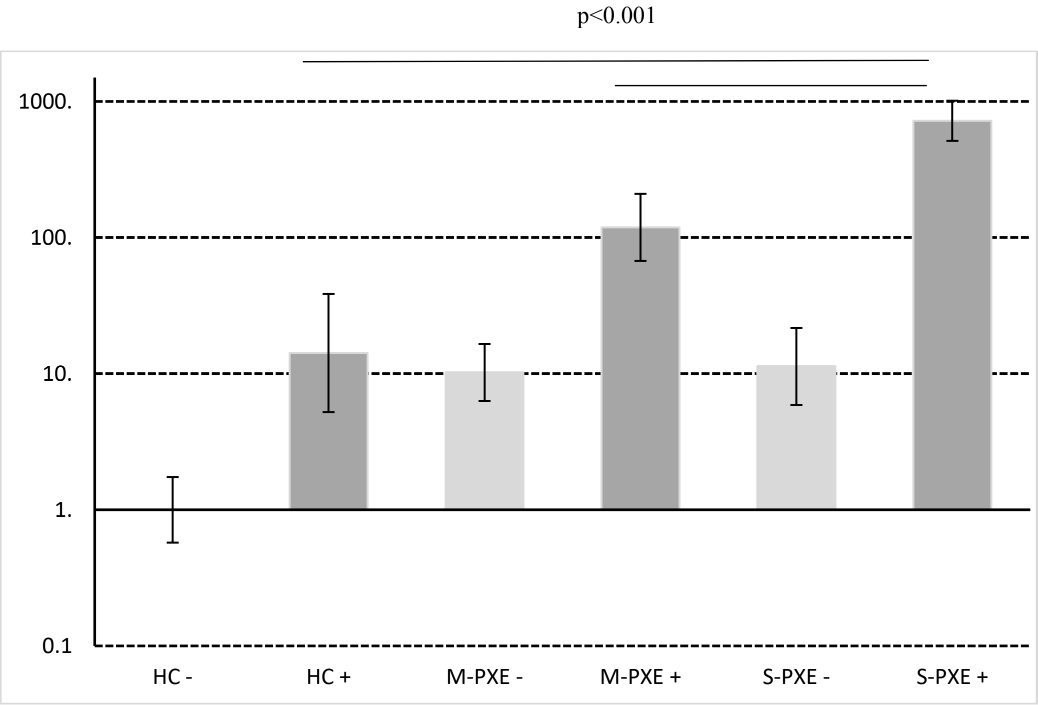

After stimulation IL1B is upregulated in all groups, confirming the validity of the experiment. In severely affected PXE patients (group S-PXE +) there was a 51-fold upregulation (p<0.001; 95%CI: 24.2-107.2) compared to an 8-fold upregulation in mild PXE patients (8-fold; p<0.001, 95%CI: 2.9-24.6). Interestingly there was a higher baseline IL1B expression in both PXE groups compared to the healthy control group (p<0.001; 95%CI: 0.04-0.21). - : fibroblasts without ILB1 stimulation, + : fibroblasts with ILB1 stimulation, HC: healthy control, M-PXE: mildly affected PXE patients, S-PXE: severely affected PXE patients.

Supplementary methods

Il1B stimulation and gene expression experiment

Patients and Cell culture

Dermal fibroblasts were used of the patients who also underwent whole exome sequencing. For each patient a skin biopsy was taken in lesional skin and primary dermal fibroblast cultures were derived. Fibroblasts were cultured using an established protocol. Additionally, anonymized dermal fibroblasts were used of two healthy Caucasian adult volunteers.

IL1B stimulation experiment

IL1B activation is a 2-step process: first, cells need to undergo a priming step in which pro-IL1B expression is stimulated using a pathogen-associated molecular pattern (PAMP); in a second step processing and secretion of active IL1B needs to be induced using a danger-associated molecular pattern (DAMP) [27]. In our validation experiment, we used LPS as a PAMP and ATP as a DAMP. At day 1 patient dermal fibroblasts were seeded in triplicate in 60x15 mm petri dishes, followed by a 48h incubation at 37°C and 5% CO_2. At day 3, medium in all petri dishes was replaced by a 1 µg/mL lipopolysaccharides (LPS, Sigma-Aldrich, Overijse, Belgium) -DMEM solution. After an incubation time of 16h, the medium in all petri dishes was renewed with a 5mM ATP (Sigma-Aldrich, Overijse, Belgium) in DMEM solution for 1 hour at 37°C/5% CO_2, followed by RNA extraction with the RNeasy mini kit according to manufacturer's guidelines (Qiagen, Antwerp, Belgium). Additionally, for each patient, dermal fibroblasts were seeded in triplicate and used as negative control.

qPCR analysis

cDNA synthesis was performed using the iScript cDNa synthesis kit (Bio-rad laboratories S.A.-N.V., Temse, Belgium), according to the manufacturer's protocol. For all primer pairs, amplification efficiencies were calculated based on a tissue-specific dilution series experiment. Quantitative PCR was performed using a LightCycler 480 (Roche, Vilvoorde, Belgium). The reaction mix consisted of 1µL 5x Master Mix (Roche, Vilvoorde, Belgium), 0.25 µL Resolight dye (Roche, Vilvoorde, Belgium), 0.25 µl of the forward and reverse primers (5µM), 2 µL DNA, supplemented to 5µL with nuclease-free water. Calculation of amplification efficiencies, reference gene stability and data analysis were performed using the qBase+ software (Biogazelle, Zwijnaarde, Belgium). The primer sequences for the *IL1B* gene as well as those for the reference genes *HPRT1* and *YWHAZ* are available upon request.

Statistics

To quantify statistical significance of differential expression, a 2-sided Mann-Whitney U test was performed (non-parametrical test, not assuming an underlying normal distribution) using the Qbase+ software. The significance level was set at 0.05.
